# Capsaicin Targets tNOX (ENOX2) to Inhibit G1 Cyclin/CDK Complex, as Assessed by the Cellular Thermal Shift Assay (CETSA)

**DOI:** 10.3390/cells8101275

**Published:** 2019-10-18

**Authors:** Atikul Islam, Ally J. Su, Zih-Ming Zeng, Pin Ju Chueh, Ming-Hung Lin

**Affiliations:** 1 Institute of Biomedical Sciences, National Chung Hsing University, Taichung 40227, Taiwan; islammiu555@gmail.com (A.I.); ally.jane.su@emory.edu (A.J.S.); jaykoyou@hotmail.com (Z.-M.Z.); 2 Department of Chemistry, Emory University, Atlanta, GA 30322, USA; 3 Graduate Institute of Basic Medicine, China Medical University, Taichung, 40402, Taiwan; 4 Department of Medical Research, China Medical University Hospital, Taichung 40402, Taiwan; 5 Division of Urology, Department of Surgery, An Nan Hospital, China Medical University, Tainan 70965, Taiwan; 6 Division of Urology, Department of Surgery, Tri-service General Hospital, Taipei 11490, Taiwan

**Keywords:** capsaicin, cell cycle, cellular thermal shift assay (CETSA), tumor-associated NADH oxidase (tNOX, ENOX2)

## Abstract

Capsaicin (8-methyl-*N*-vanillyl-6-noneamide), which is an active component in red chili peppers, is used as a chemopreventive agent that shows favorable cytotoxicity against cancer cells. Accumulating evidence indicates that capsaicin preferentially inhibits a tumor-associated NADH oxidase (tNOX, ENOX2) that is ubiquitously expressed in cancer but not in non-transformed cells. This attenuates cancer cell growth by inducing apoptosis. The capsaicin-mediated inhibition of tNOX was recently shown to prolong the cell cycle. However, the molecular events underlying this regulation have not yet been investigated. In the present study, we used a cellular thermal shift assay (CETSA) to detect “target engagement” of capsaicin and its consequent impact on cell cycle progression. Our results indicated that capsaicin engaged with tNOX and triggered the proteasomal degradation of tNOX, which leads to the inhibition of NAD^+^-dependent SIRT1 deacetylase. Ultimately, the acetylation levels of c-Myc and p53 were enhanced, which suppressed the activation of G1 cyclin/Cyclin-dependent kinase complexes and triggered cell cycle arrest in cancer cells. The results obtained when tNOX was overexpressed in non-cancer cells validated its importance in cell cycle progression. These findings provide the first molecular insights into the regulatory role of tNOX and the anti-proliferative property of capsaicin in regulating the cell cycle of bladder cancer cells.

## 1. Introduction

Capsaicin (8-methyl-*N*-vanillyl-6-noneamide) is a common component of the chili pepper that causes the sensation of burning. It has been commonly used as a food additive and medically applied as an analgesic [1]. However, accumulating evidence suggests that capsaicin possesses anti-growth activity against various cancer cell systems, and, thus, has the potential to act as a chemo-preventive agent [2,3]. Apoptosis induction has been well explored as a major mechanism of capsaicin-mediated cytotoxicity. However, cell cycle regulation and the consequent regulation of cell proliferation has also been studied in the anti-proliferative properties for human colon [4], breast [5], KB [6], and bladder [7] cancer cells. The central proteins involved in regulating the cell cycle are the cyclins, cyclin-dependent kinases (CDKs), and CDK inhibitors. Activation of the cyclin/CDK complex provides the driving force to push cells from one phase to the next. For example, cyclin D and cyclin E1 are known as proto-oncogenes. Along with their counterpart CDKs, they can suppress the retinoblastoma gene whose product inhibits the cell cycle. This allows cells to progress from the G1 phase to the S phase and prevent excessive growth. Capsaicin has been shown to induce cell cycle arrest by inhibiting CDK2, CDK4, and CDK6 in bladder cancer cells [8], but the molecular mechanisms underlying this effect remain unknown.

We previously demonstrated that capsaicin preferentially inhibits a tumor-associated NADH oxidase (tNOX, ENOX2) in cancer/transformed cells, and, thereby, enhances ROS generation and apoptosis [9,10,11,12]. tNOX belongs to a family of growth-related NADH (or hydroquinone) oxidases whose members can convert reduced NADH to oxidized NAD^+^ [13,14,15]. Results obtained from experiments involving the depletion of tNOX in cancer cells and its overexpression in non-cancer cells indicate that tNOX is involved in cell growth regulation [10,16,17,18]. However, compared to the plentiful results demonstrating the association between tNOX and apoptosis, much less is known regarding the function of tNOX in regulating the cell cycle. A recent study provided evidence supporting the idea that the capsaicin-mediated inhibition of tNOX is involved in cell cycle regulation [19]. In this case, we set out to further reveal the action mechanism of capsaicin. Using a cellular thermal shift assay (CETSA) in T24 bladder cancer cells, we show that capsaicin directly engages with tNOX, and that this binding inhibits NAD^+^ generation and downregulates tNOX, which suppresses sirtuin-1 (SIRT1) deacetylase activity. Ultimately, this decrease in SIRT1 deacetylase activity contributes to a cell cycle arrest through enhanced acetylation of c-Myc and p53. This suppresses cyclins D and E, which are both essential for the ability of the cell to move from the G1 phase to the S phase.

## 2. Materials and Methods

### 2.1. Cell Culture and Reagents

Capsaicin (purity >95%) was purchased from Sigma-Aldrich Corporation (St. Louis, MO, USA). The anti-SIRT1, anti-p21, anti-p53, anti-acetyl-p53, anti-phosphorylated Rb, anti-cyclin D1, anti-cyclin E2, and anti-phospho-ERK antibodies were purchased from Cell Signaling Technology, Inc. (Beverly, MA, USA). The anti-β-actin, anti-p27, and anti-acetyl-c-Myc antibodies were from Millipore Corp. (Temecula, CA, USA). The anti-CDK4, anti-c-Myc, anti-E_2_F_1_, and anti-GST antibodies were purchased from Santa Cruz Biotechnology, Inc. (Santa Cruz, CA, USA). The anti-ERK antibody was from BD Biosciences (San Jose, CA, USA). The antisera to tNOX were generated as described previously [20]. The anti-mouse and anti-rabbit IgG antibodies and other chemicals were purchased from the Sigma Chemical Company (St. Louis, MO, USA), unless otherwise specified.

T24 human bladder carcinoma cells were grown in RPMI and NIH3T3 (mouse fibroblast) cells that were grown in DMEM. Media were supplemented with 10% fetal bovine albumin, 100 units/mL penicillin, and 50 µg/mL streptomycin. Cells were maintained at 37 °C in a humidified atmosphere of 5% CO_2_ in air, and the media were replaced every 2 to 3 days. Cells were treated with different concentrations of capsaicin (dissolved in ethanol), as described in the text, or with the same volume of ethanol (vehicle control). NIH3T3 cells were transiently transfected with wild type or mutant forms of tNOX-GST or GST (control vector) using the jetPEI transfection reagent, according to the manufacturer’s protocol (Polyplus-transfection SA, Illkirch Cedex, France), as described previously [18].

### 2.2. Cell Impedance Measurements

Cell impedance technology was used to continuously monitor changes in cell growth. Cells (10^4^ per well) were seeded onto E-plates and incubated for 30 min at room temperature, and the E-plates were placed onto the xCELLigence System (Roche, Mannhein, Germany). Cells were grown overnight before being exposed to ethanol or different concentrations of capsaicin. Cell impedance was measured every hour, as previously described [21].

### 2.3. Cell Viability Assays

Cells were seeded onto 96-well culture plates at 8 × 10^3^ cells per well and permitted to adhere overnight at 37 °C. After incubation, cells were treated with a 0.5 mg/mL solution of 3-(4,5-dimethylthiazolyl-2)-2,5-diphenyltetrazolium bromide (MTT, 20 μL/well) for 3 h at 37 °C. The number of viable cells was determined by the uptake of MTT, assayed at 495 nm. All experiments were performed at least in triplicate on three separate occasions. Data are presented as mean ± S.D.

### 2.4. Cell Cycle Analysis

In brief, after treatments, 10^6^ cells were collected and washed in PBS, slowly fixed in 75% ethanol, and kept at −20 °C overnight. The cell pellet was then washed with PBS, and centrifuged at 500× *g* for 5 min. The pellet was resuspended in 200 μL cold PBS, and the nuclear DNA was stained with a propidium iodide (PI) solution (20 mM Tris pH 8.0, 1 mM NaCl, 0.1% NP-40, 1.4 mg/mL RNase A, 0.05 mg/mL PI) for 30 min on ice in the dark. The total cellular DNA content was analyzed with a FC500 flow cytometer (Beckman Coulter Inc., Brea, CA, USA).

### 2.5. Cellular Thermal Shift Assay (CETSA)

Engagement between capsaicin and tNOX in cells was analyzed by cellular thermal shift assay. Samples were prepared from control and drug-exposed cells. For each set, 2 × 10^7^ cells were seeded in a 10-cm cultured dish. After 24 h of culturing, the cells were pretreated with 10 μM MG132 for 1 h, washed with PBS, treated with trypsin, and collected. The samples were centrifuged at 12,000 rpm for 2 min at room temperature, the pellets were gently resuspended with 1 mL of PBS, and the samples were centrifuged at 7500 rpm for 3 min at room temperature. The pellets were resuspended with 1 mL of PBS containing 20 mM Tris-HCl pH 7.4, 100 mM NaCl, 5mM EDTA, 2 mM phenylmethylsulfonyl fluoride (PMSF), 10 ng/mL leupeptin, and 10 μg/mL aprotinin. The samples were transferred to Eppendorf tubes and subjected to three freeze-thaw cycles. For each cycle, they were exposed to liquid nitrogen for 3 min, placed in a heating block at 25 °C for 3 min, and vortexed briefly. The samples were then centrifuged at 12,000 rpm for 30 min at 4 °C, and the supernatants were transferred to new Eppendorf tubes. For the experimental sample set, capsaicin was added to a final concentration of 2 mM. For the control sample set, the same volume of vehicle solvent was added. The samples were heated at 25 °C for 1 h and dispensed to 100 μL aliquots. Pairs consisting of one control aliquot and one experimental aliquot were heated at 43 °C, 46 °C, 49 °C, 52 °C, 55 °C, 58 °C, 61 °C, or 65 °C for 3 min. Lastly, the samples were placed on ice and subjected to Western blot analysis using antisera raised against tNOX.

### 2.6. Determination of the Cell-Doubling Time

Cells exposed to different concentrations of capsaicin were labeled by incubation with 5 μM CellTracker Green CMFDA (5-chloromethylfluorescein diacetate, Molecular Probes, Eugene, OR, USA) in fresh medium for 45 min. The cells were then collected by trypsinization and centrifugation, washed with PBS, centrifuged at 200× *g* for 5 min, and analyzed immediately using a Beckman Coulter FC500 flow cytometer.

### 2.7. Western Blot Analysis

Cell extracts were prepared in lysis buffer containing 20 mM Tris-HCl pH 7.4, 100 mM NaCl, 5 mM EDTA, 2 mM phenylmethylsulfonyl fluoride (PMSF), 10 ng/mL leupeptin, and 10 μg/mL aprotinin). Volumes of extract containing equal amounts of proteins (40 µg) were applied to SDS-PAGE gels, and resolved proteins were transferred to nitrocellulose membranes (Schleicher & Schuell, Keene, NH, USA). The membranes were blocked with nonfat milk solution for 30 min, washed, and probed with a primary antibody. The membranes were then rinsed with Tris-buffered saline containing 0.1% Tween 20, and incubated with a horseradish peroxidase-conjugated secondary antibody for 2 hours. The membranes were rinsed again and developed using enhanced chemiluminescence (ECL) reagents (Amersham Biosciences, Piscataway, NJ, USA). The intensity of the tNOX protein band was quantified using Gel-pro analysis 3.1 software. The obtained values were normalized to those obtained for actin.

### 2.8. Statistics

All data are expressed as the mean ± SD of three or more independent experiments. Comparison between groups was made by one-way analysis of variance (ANOVA) followed by an appropriate post-hoc test, such as LSD or the t-test. A value of *p* < 0.05 was considered to be statistically significant.

## 3. Results

### 3.1. CETSA Shows that There is a Binding Interaction Between Capsaicin and tNOX

**Figure 1:**
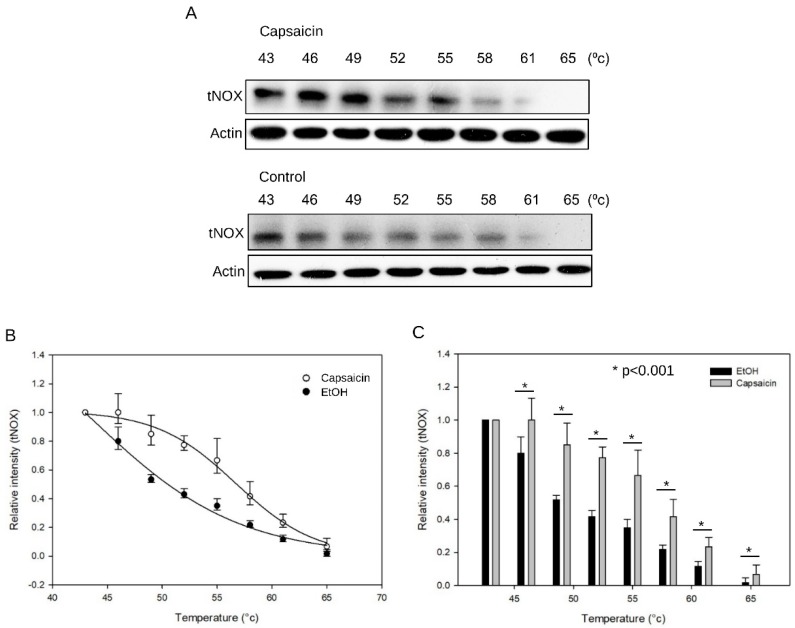
CETSA-based determination of binding between capsaicin and tNOX. (**A**) CETSA was performed as described in the Materials and Methods section. Cell lysates were separated by SDS-PAGE and analyzed by Western blotting. β-Actin was detected as an internal control. Representative images are shown. (**B**) CETSA curves of tNOX in T24 cells were determined in the absence and presence of capsaicin. Each band intensity of tNOX was normalized with respect to that obtained at 43 °C. The graphs are an average of three independent experiments. (**C**) The quantification of relative intensity of tNOX protein versus increased temperature from three independent experiments (* *p* < 0.001).

Evidence has indicated that tNOX is involved in various capsaicin-induced cellular responses, including apoptosis and changes in cell migration [10,19,22]. However, it remained unclear whether tNOX is a direct target of capsaicin. To determine whether capsaicin directly binds to tNOX, we used CETSA to perform label-free target validation, which is based on the idea that ligand binding enhances the thermal stability of a target protein [23,24]. We found that, when T24 cell lysates were incubated with capsaicin, the thermal stability of tNOX was increased when compared to the control group (Figure 1A). We plotted the relative tNOX protein against temperatures to generate thermal melting curves, and used them to calculate melting temperatures (*T*_m_, the temperature at which 50% of proteins are unfolded and rapidly precipitated by heat). We observed a noticeable difference in the curves of controls compared to capsaicin-treated lysates of T24 cells. The average *T*_m_ value increased from 48.5 °C in the control group to 56.7 °C in the capsaicin-treated group, which indicates capsaicin-triggered thermal stabilization of tNOX through direct binding (Figure 1B,C). 

### 3.2. Capsaicin-Mediated Inhibition of tNOX Inhibits SIRT1 to Enhance the Acetylation of p53 and c-Myc

**Figure 2:**
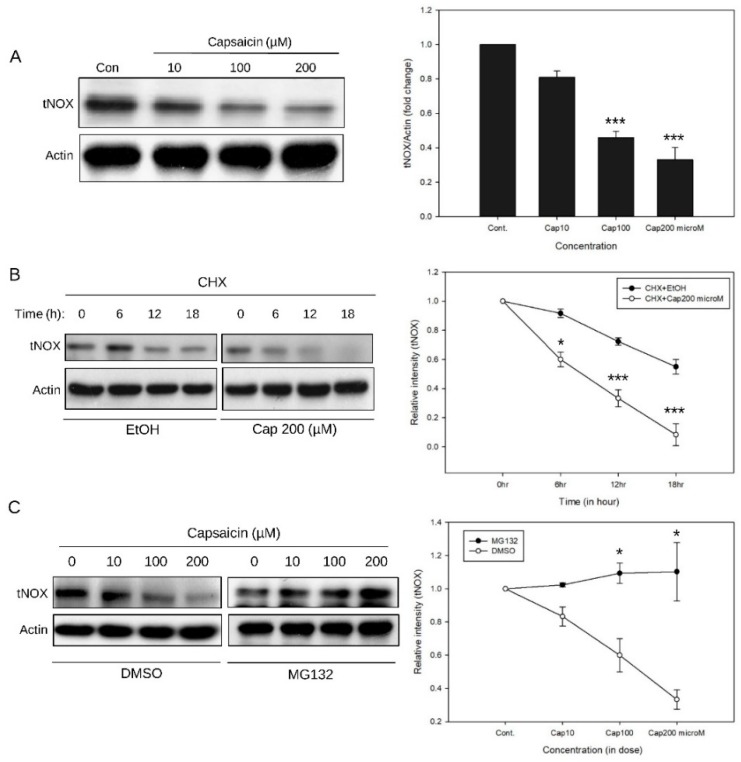
Capsaicin suppresses tNOX expression through proteasome-mediated degradation. Aliquots of cell lysates were separated by SDS-PAGE and analyzed by Western blotting. β-Actin was detected as an internal control. Representative images are shown. Values (mean ± S. E.) are from at least three independent experiments (* *p* < 0.05, *** *p* < 0.001 for capsaicin-treated cells vs. controls). (**A**) tNOX expression was significantly decreased by capsaicin at 100 and 200 μM. (**B**) Capsaicin (200 μM) clearly reduces tNOX stability as assessed with a cycloheximide-chase assay. (**C**) The proteasome inhibitor, MG132, reverses the capsaicin-induced suppression of tNOX expression.

We next examined the effect of capsaicin on tNOX protein expression. Consistent with previous studies, our data confirmed that capsaicin markedly and dose-dependently suppressed the tNOX protein expression of T24 cells (Figure 2A). Using a cycloheximide-chase assay, we were able to show that 200 μM capsaicin markedly reduced the half-life of tNOX in T24 cells starting at 6 h (Figure 2B). Treatment with the proteasome inhibitor, MG132, significantly enhanced the stability of tNOX in T24 cells exposed to capsaicin, which indicates that proteasomal degradation was involved in the capsaicin-induced suppression of tNOX expression (Figure 2C).

**Figure 3:**
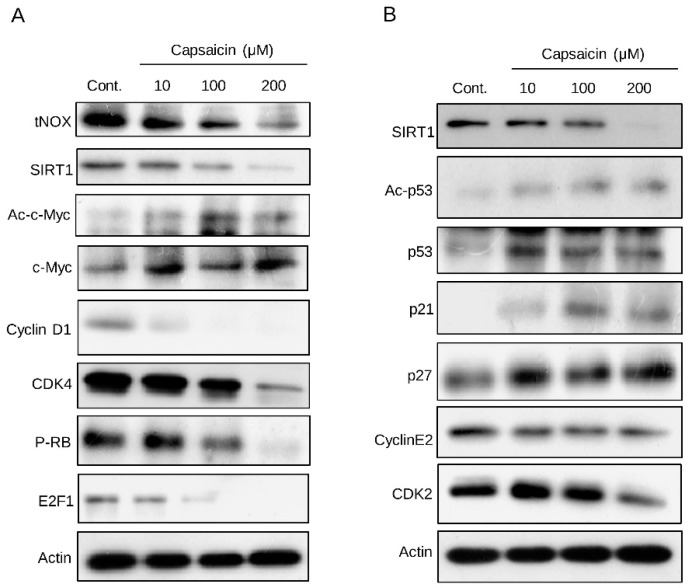
Capsaicin induces cell cycle arrest at the G0/G1 phases. T24 cells were treated with capsaicin or ethanol for 24 h. Aliquots of cell lysates were separated by SDS-PAGE and analyzed by Western blotting. β-Actin was detected as an internal control. Representative images are shown.

**Figure 4:**
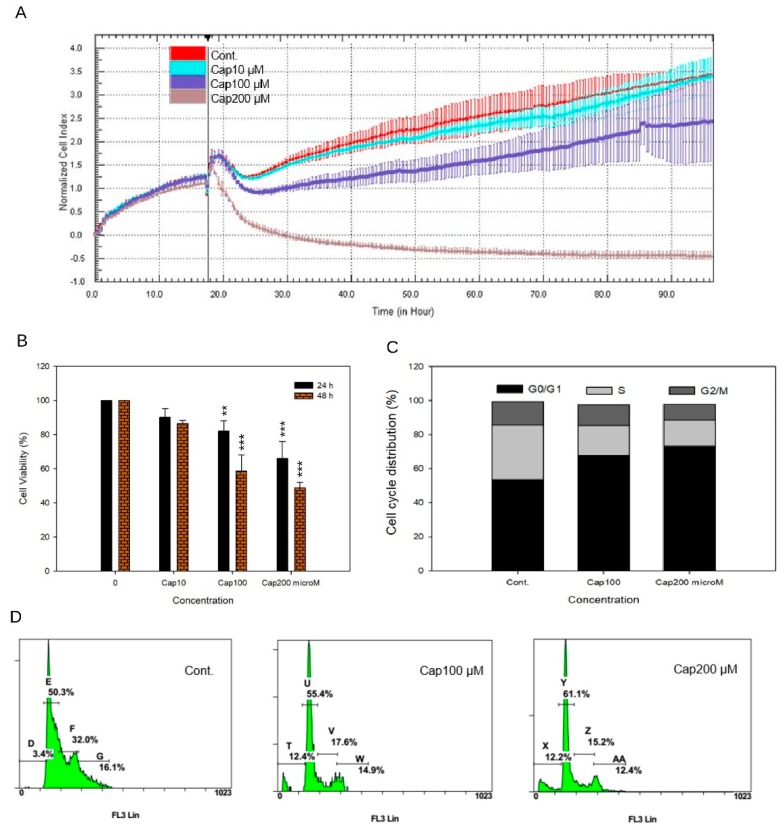
Effect of capsaicin on the proliferation and cell cycle of T24 cells. (**A**) Dynamic monitoring of cell proliferation was performed using impedance technology, as described in the Materials and Methods section. Normalized cell index values measured over 96 h are shown. (**B**) Cells were exposed to different concentrations of capsaicin for 24 or 48 h and cell viability was measured using MTT assays. Values (means ± SDs) are from three independent experiments. There was a significant decrease in cell viability in cells treated with capsaicin when compared with controls (** *p* <0.01, *** *p* <0.001). (**C**,**D**) T24 cells were exposed to different concentrations of capsaicin for 24 h and flow cytometry was used to assess their cell-cycle phase. The graphs are representative of three independent experiments.

We next examined the cellular outcomes of the ability of capsaicin to bind tNOX and suppress its expression. In cancer cells, the capsaicin-triggered inhibition of tNOX was previously shown to decrease the intracellular NAD^+^/NADH ratio and suppress NAD^+^-dependent sirtuin 1 (SIRT1) deacetylase activity [21]. In this case, we observed that treatment of T24 cells with 100 or 200 μM capsaicin reduced SIRT1 expression and increased c-Myc acetylation (Figure 3A). The latter change prevented c-Myc from interacting with Max. This attenuated the ability of Myc-Max to transcriptionally activate the expression of cyclin D1, which is important for the G1/S transition (Figure 3A). We also observed that capsaicin enhanced the acetylation/activation of p53, which leads to the up-regulation of p21 and p27 (Figure 3B). The up-regulation of p21, which is a CDK inhibitor, suppressed the activation of the cyclin E/CDK2 complex, which obstructed the phosphorylation of pRb and its subsequent activation of E_2_F_1_ (Figure 3). These changes are suggestive of a cell cycle arrest at the G0/G1 phases. The capsaicin-induced inhibition of cyclin D/CDK4 and cyclin E/CDK2 both contributed to the inactivation of E_2_F_1_, which is important for subsequent S phase entry. Collectively, our results suggest that capsaicin treatment of T24 cells arrests the cell cycle at the G0/G1 phases (Figure 3). The cellular impact of capsaicin was also examined using the xCELLigence System, which is a label-free, real-time cell monitoring platform that measures electrical impedance and displays the results as cell index values. Our data confirmed that capsaicin dose-dependently inhibits the growth of T24 bladder cancer cells (Figure 4A). Cell viability assays were also performed to validate the inhibitory effect of capsaicin on T24 cells. Capsaicin significantly reduced cell viability in a dose-dependent and time-dependent manner (Figure 4B). To further validate the anti-proliferative effect of capsaicin, we analyzed changes in the cell cycle distributions. Our results demonstrated that capsaicin triggered a significant accumulation ofT24 cells in the G0/G1 phase (Figure 4C,D).

### 3.3. Overexpression of tNOX in Non-Cancer Cells that Have a Shorter Cell Doubling Time and Enhanced Cell Proliferation

**Figure 5:**
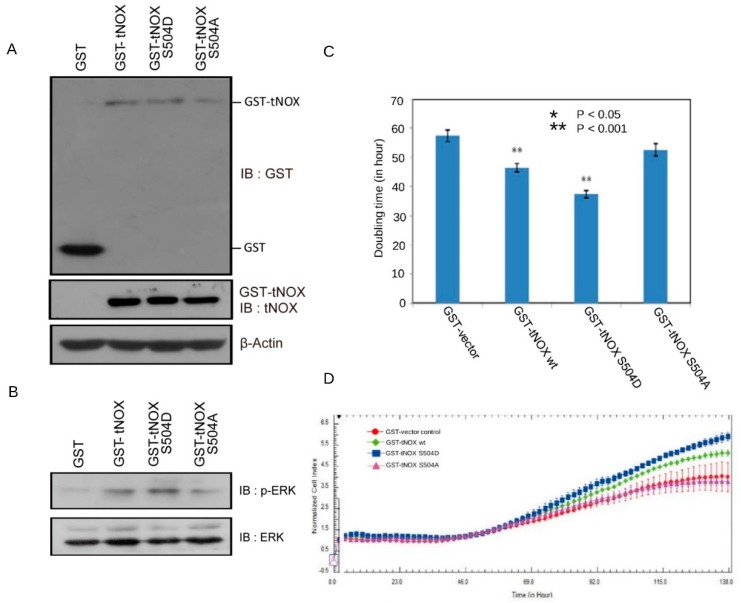
tNOX overexpression in NIH3T3 cells enhances cell growth. NIH3T3 cells were transfected with GST, GST–tNOX wild-type, GST–tNOX^S504D^, or GST–tNOX^S504A^. (**A**,**B**) Overexpression of GST–tNOX wild-type, GST–tNOX^S504D^, or GST–tNOX S504A fusion proteins was analyzed using anti-GST, anti-tNOX, anti-phospho-ERK, and anti-ERK antibodies. β-Actin was detected as a loading control. Representative images are shown. (**C**) The cell-doubling time was determined by CMFDA staining of NIH3T3 cells. The presented values (mean ± S.E.) represent at least three independent experiments (** *p* < 0.001). (**C**) Dynamic monitoring of cell proliferation was performed using impedance technology, as described in the Materials and Methods section. Normalized cell index values measured over 138 h are shown.

To confirm the ability of tNOX to regulate the cell cycle, we examined the cellular impact of tNOX overexpression in NIH3T3 cells transfected with GST-fused wild-type tNOX or its mutant versions, tNOX^S504D^ and tNOX^S504A^ (Figure 5A). Cells overexpressing wild-type tNOX and tNOX^S504D^ exhibited higher levels of phosphorylated-ERK, which suggests an enhancement of cell proliferation (Figure 5B). Analysis of the cell division ability (i.e., the cell doubling time) revealed that, after 2 days in culture, wild-type tNOX and tNOX^S504D^-overexpressing NIH3T3 cells displayed a higher mean CellTracker Green CMFDA fluoresce compared to the GST-vector controls (Figure 5C). This indicates that the cell doubling time was shortened, which contributed to accelerating the cell cycle progression. Overexpression of the tNOX^S504A^ mutant did not significantly impact the cell doubling time compared to that in the mock controls (Figure 5C). This would seem to emphasize the previously reported functional importance of serine-504 phosphorylation in tNOX [18]. Similar results were obtained when we used the xCELLigence System to continuously monitor the growth of tNOX-overexpressing non-cancer cells. Using this approach, we found that overexpression of wild-type tNOX and the tNOX^S504D^ mutant in NIH3T3 cells markedly enhanced cell proliferation compared to the vector control and the tNOX^S504A^ mutant (Figure 5D). On the basis of our present data, we conclude that tNOX plays a regulatory role in cell cycle progression and its engagement with capsaicin affects its activity and expression, which ultimately leads to cell cycle arrest at the G0/G1 phase.

## 4. Discussion

Capsaicin has long been considered a chemopreventive agent, but recent evidence suggests that it may also modulate an array of signaling pathways. These pathways produce cellular outcomes ranging from cell death to cell survival. There is much that remains unknown regarding the cellular targets of capsaicin and the molecular mechanisms initiated by this agent. Many research groups, including ours, have explored the molecular targets and signaling pathways involved in the anti-cancer properties of capsaicin. Capsaicin has been shown to induce G0/G1 cell cycle arrest by activating p53 and p21 and subsequent inhibition of cyclin E and CDK2 in human esophageal epidermoid carcinoma CE 81T/VGH cells [25]. In another study, capsaicin-induced G0/G1 cell cycle arrest was demonstrated to act by inhibiting cyclin-dependent kinases, such as CDK2, CDK4, and CDK6 [26]. However, there is little direct physical evidence regarding the targets of capsaicin, and there has been little discussion of the molecular events underlying the capsaicin-induced induction of p53/p21 or inhibition of cyclin-dependent kinases. In this case, we used CETSA to show for the first time that tNOX (a tumor-associated NADH oxidase) is a cellular target of capsaicin. The engagement with capsaicin affected the stability of tNOX and its function in cell cycle regulation by reducing the generation of NAD^+^ and the inhibition of SIRT1. This enhanced the acetylation of c-Myc and p53 and, thereby, inactivated the cyclin/CDK complexes that are important for the ability of the cell to leave the G1 phase and enter the S phase. The identification of tNOX as a capsaicin-binding target, thus, provides important new insights into the mechanisms underlying the efficacy of capsaicin.

Recent progress has shed light on the differential cellular responses induced by capsaicin in cancer and non-cancer cells and provided insight into the long-standing dispute regarding the cellular target(s) of capsaicin [27,28,29,30]. In cancer/transformed cells, which exhibit relatively high-level expression of tNOX, capsaicin preferentially inhibits tNOX activity to oxidize hydroquinones and NADH (to the NAD^+^ form), which results in apoptosis induction and growth reduction. In a non-cancerous cell, in contrast, tNOX expression is low and capsaicin has relatively little effect [10,12,14]. Our group further clarified that, in cancer, A549 cells reduced tNOX-NAD^+^ generation and modulated NAD^+^-dependent SIRT1 deacetylase to increase p53 acetylation, which results in enhanced cytotoxic apoptosis whereas, in noncancerous MRC-5 cells, it increased the NAD^+^/NADH ratio and SIRT1 deacetylase activity toward Atg5. This leads to survival-promoting autophagy [21]. In the present work, we explored whether capsaicin could attenuate cell proliferation, with a focus on the tNOX-mediated and SIRT1-mediated regulation of the cell cycle. We found that SIRT1 contributes to the capsaicin-induced cell cycle arrest through tNOX-inhibited NAD^+^ generation. SIRT1 is a member of the sirtuin protein family of NAD^+^-dependent deacetylases. The members of this family are responsible for post-translationally modifying histones and nonhistone proteins [31,32]. Few previous studies have examined the association between capsaicin and SIRT1, and even fewer have assessed their role in growth regulation [33,34,35]. We previously reported that tNOX knockdown in T24 cells reversed cancer phenotypes in association with downregulation of SIRT1 and cyclin D1, even though the details of the underlying mechanism remained unclear [19]. We speculate that tNOX-depletion activates p53 to transcriptionally down-regulate microRNA-34a, which negatively regulates the SIRT1 protein [36,37,38]. Based on the previous and present findings, we propose that capsaicin targets tNOX to alter its enzymatic activity and stability. This down-regulation of tNOX along with concurrent SIRT1 deacetylase inhibition increases the acetylation of c-Myc and p53 to repress key molecules that are important for cell cycle progression, such as cyclin D, cyclin E, and E_2_F. Our present results provide significant evidence that tNOX functions as a direct target of capsaicin, and that this could reasonably explain the ability of capsaicin to induce cell cycle arrest at the G0/G1 phases.

The effectiveness of a drug typically depends on the binding of its anticipated target in cells. Thus, we must identify the protein targets of capsaicin if we hope to understand its mechanisms of action. Although the accumulated data indicate that tNOX contributes to the anti-cancer properties of capsaicin, it was previously unknown whether this involved direct binding between capsaicin and tNOX. To analyze target engagement in live cells, we used CETSA, which can quantify the engagement between an endogenous target protein and an unlabeled drug by monitoring ligand-induced protein stabilization at a fixed ligand concentration under increasing temperature [23,24]. CETSA has become one powerful label-free method to assess target engagement in the physiological environment. For example, CETSA can be reliably utilized to understand the mechanism of action of the drug despite the engagement of small molecules to membrane protein targets of different complexities [39]. The feasibility of CETSA is also validated in the detection of physiologically important transmembrane protein-solute carriers, SLC1A2 and SLC16A1 [40]. Using this approach, we detected the remaining levels of soluble tNOX protein by quantitative Western blot analysis using anti-tNOX antisera. Our results provide the first physical and direct evidence that tNOX is a cellular target of capsaicin. We recently reported that the engagement between tNOX and oxaliplatin, which is a platinum-based drug that is used clinically to treat cancer, contributes to inhibiting tNOX and inducing apoptosis in colon cancer cells [41].

## 5. Conclusions

Taken together, our current identification of tNOX as a drug target both improves our understanding of the action mechanisms of anti-cancer drugs and suggests that tNOX may have therapeutic applications, such as the ability to prolong the cell cycle of cancer cells.
